# Impact of Male Circumcision on the HIV Epidemic in Papua New Guinea: A Country with Extensive Foreskin Cutting Practices

**DOI:** 10.1371/journal.pone.0104531

**Published:** 2014-08-11

**Authors:** Richard T. Gray, Andrew Vallely, David P. Wilson, John Kaldor, David MacLaren, Angela Kelly-Hanku, Peter Siba, John M. Murray, on behalf of the Male Circumcision Acceptability and Impact Study (MCAIS) team

**Affiliations:** 1 The Kirby Institute, University of New South Wales, Sydney, New South Wales, Australia; 2 Sexual and Reproductive Health Unit, Papua New Guinea Institute of Medical Research, Goroka, Eastern Highlands, Papua New Guinea; 3 School of Medicine and Dentistry, James Cook University, Cairns, Queensland, Australia; 4 International HIV Research Group, School of Public Health and Community Medicine, University of New South Wales, Sydney, New South Wales, Australia; 5 School of Mathematics and Statistics, University of New South Wales, Sydney, New South Wales, Australia; University of Melbourne, Australia

## Abstract

The degree to which adult medical male circumcision (MC) programs can reduce new HIV infections in a moderate HIV prevalence country like Papua New Guinea (PNG) are uncertain especially given the widespread prevalence of longitudinal foreskin cuts among adult males. We estimated the likely impact of a medical MC intervention in PNG using a mathematical model of HIV transmission. The model was age-structured and incorporated separate components for sex, rural/urban, men who have sex with men and female sex workers. Country-specific data of the prevalence of foreskin cuts, sexually transmitted infections, condom usage, and the acceptability of MC were obtained by our group through related studies. If longitudinal foreskin cutting has a protective efficacy of 20% compared to 60% for MC, then providing MC to 20% of uncut males from 2012 would require 376,000 procedures, avert 7,900 HIV infections by 2032, and require 143 MC per averted infection. Targeting uncut urban youths would achieve the most cost effective returns of 54 MC per HIV infection averted. These numbers of MC required to avert an HIV infection change little even with coverage up to 80% of men. The greater the protective efficacy of longitudinal foreskin cuts against HIV acquisition, the less impact MC interventions will have. Dependent on this efficacy, increasing condom use could have a much greater impact with a 10 percentage point increase averting 18,400 infections over this same period. MC programs could be effective in reducing HIV infections in PNG, particularly in high prevalence populations. However the overall impact is highly dependent on the protective efficacy of existing longitudinal foreskin cutting in preventing HIV.

## Introduction

Based on randomized trials in sub-Saharan Africa, adult medical male circumcision (MC) reduces HIV acquisition in men during heterosexual intercourse by approximately 60% [1]–[3]. In high HIV prevalence countries, mathematical modeling has predicted that increased MC could substantially reduce HIV incidence [4]–[7]. The intervention is considered moderately cost-effective requiring between 5 and 15 MC at a cost of $US150 to $US900 to avert an HIV infection [6], although other calculations produce higher estimates of 19 to 58 MCs per infection averted [8]. Based on this evidence, voluntary MC has become a priority prevention intervention by global funders and countries in southern and eastern Africa [9], [10]. Countries in this region have high adult HIV prevalence [11] and it is unknown if the benefits of MC translate to countries with relatively low HIV prevalence but containing populations and regions with elevated HIV levels, such as Papua New Guinea (PNG).

**Figure 1:**
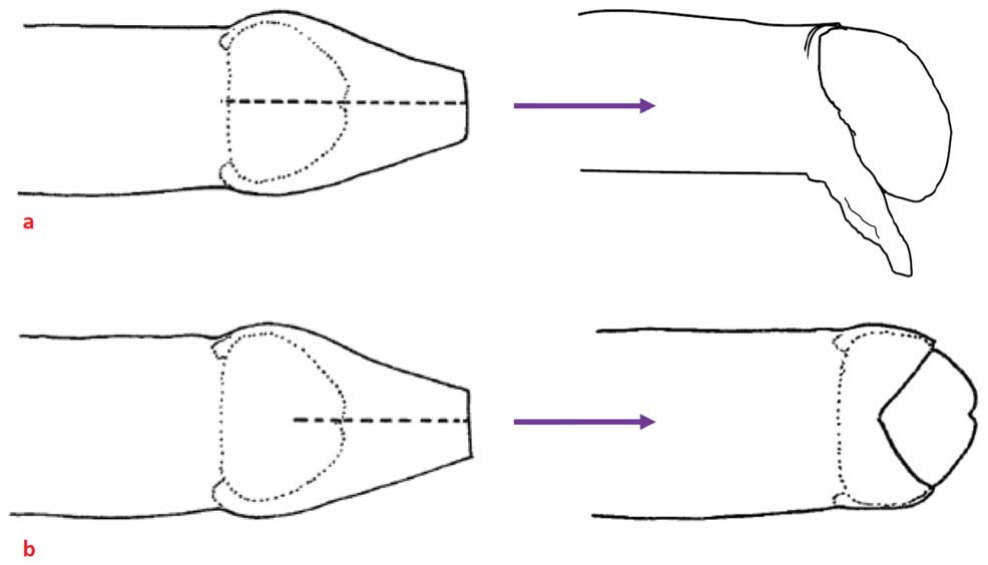
Diagrams of the most common penile cuts in PNG. “a) longitudinal dorsal slit, complete: resulting in lateral retraction of the foreskin and a flap of skin that hangs down below the penis; b: longitudinal dorsal slit, incomplete: resulting in V shaped opening” [16]. Reproduced with permission from Kelly et al. [16].

The possible impact of MC programs is also affected by acceptability of MC in the particular cultural setting as well as any pre-existing foreskin cutting practices. In Africa these practices traditionally involve circumcising the foreskin to varying extents which may then differentially affect the likelihood of HIV acquisition [12], [13]. However in other regions male foreskin cutting can be much more varied and rarely involve circumcision. In PNG, dorsal longitudinal foreskin cuts (‘straight cut’ or dorsal slit) are much more common than full removal of the foreskin (‘round cut’) [14]. In dorsal longitudinal cut procedures, the foreskin is cut along the dorsal surface and initially hangs below the ventral surface but subsequently appears to retract to form a skin remnant around the ventral and lateral aspects of the base of the glans. Following a longitudinal cut the glans is exposed to varying degrees dependant on the extent of the cut (Figure 1) [15], [16]. A longitudinal cut may confer some degree of protection against HIV acquisition in men in a similar way to full removal of the foreskin, because the lateral retraction and eversion of the foreskin that follows a dorsal slit result in exposure of the glans and inner foreskin, and a final appearance that can closely resemble medical circumcision.

In a recent study of over 800 men across four sites in PNG, 47% of men had some form of longitudinal cut compared to 10% with complete foreskin removal [14]. Only 3.4% of male plantation workers in PNG reported full circumcision compared to 25.8% who reported a dorsal slit [17]. The minority of foreskin cutting that involves complete removal of the foreskin in PNG contrasts markedly with Xhosa men in Cape Town, South Africa where 90.5% of men with traditional circumcision had the foreskin completely removed [13]. Assessing the impact of this pre-existing level of foreskin cutting in PNG is therefore more complicated than in the African setting. Acceptability of MC programs however is high in PNG making the question of whether they would be effective even more pressing.

In two related acceptability investigations of MC across eight sites in PNG, a majority of men were in favor of MC being introduced for HIV prevention [14], [18], but that MC acceptability among women was more variable in part because of concerns about sexual risk compensation [19]. Uncut men who favoured MC overwhelmingly stated they wished the procedure to take place in a clinical setting if/when they had the procedure because of a perception that it was a safer place to have the procedure performed. There are considerable risks associated with foreskin cutting performed outside a clinical setting, and the majority of foreskin cutting is either self-performed or among a group of peers in teenage men. It is performed by extending the foreskin, inserting a spatula (often a wooden ice-cream stick) below the foreskin to protect the glans, and then cutting along a line above the spatula often with a razor blade or a scalpel blade obtained from a health centre [15], [16] (Figure 1). In some rural settings dried and hardened bamboo or shells are used for the cutting instrument. This and other more extensive cutting procedures, such as the traditional form 'V cut', can have significant complications, including excessive bleeding and infection from unsterilized implements [15]. The lack of facilities (and/or options within the facilities) influenced the amount of cutting in a community setting. Whether MC is implemented or not, there is currently a high prevalence of foreskin cutting among sexually active men in PNG with unknown benefit towards lowering HIV risk [14], [16], and a burden associated with complications from these procedures performed outside appropriate facilities.

These non-MC forms of foreskin cutting have an unknown efficacy in the reduction of acquiring HIV, but health system professionals report an increasing frequency especially in adolescent males, with foreskin cutting occurring prior to sexual debut [15], [16]. Furthermore, foreskin cutting is often performed away from counseling services and outside of a clinic setting resulting in health complications [14], [20]. These factors complicate assessments of the additional benefits provided by population-based MC programs in PNG.

Papua New Guinea (PNG) is a developing country in the Asia Pacific region with an adult HIV prevalence of 0.7% in 2011 [21]. The drivers of the HIV epidemic in PNG are similar to those of the epidemic in sub-Saharan Africa. HIV is spread predominantly through heterosexual transmission, there is a higher prevalence in women than men, and women are diagnosed at an earlier age than men [22]. High community levels of sexually transmitted infections (STI) (with prevalence of approximately 20% for *Chlamydia trachomatis* and HSV-2 [23]), reflect low condom usage, poor access to medical services, and an increased risk of HIV infection [24].

In collaboration with Australian and PNG-based research institutes, the PNG National Department of Health, other government stakeholders and development partners in PNG, we investigated issues related to MC including the acceptability of MC in PNG [18], the types of foreskin cutting performed [16], and the resulting health complications [15], [25]. The results of this work contributed to the development of a PNG-relevant mathematical model of the HIV epidemic, along the lines of previous models used to assess the impact of MC in sub-Saharan Africa by our group and others [4], [5]. We used this model to investigate the potential impact of MC interventions in PNG and the impact of non-MC forms of foreskin cutting.

## Methods

### Summary of the PNG HIV Model

We developed an age-structured mathematical model based on methodology we had previously used in sub Saharan African modelling [5], but incorporating features characteristic of the PNG setting. Specifically, we included rural and urban populations, the impact of high STI prevalences as a cofactor in HIV transmission/acquisition [24], [26], [27], a separate male group who engage in both homosexual and heterosexual intercourse (as evident in PNG [28], [29]), and a higher HIV prevalence female sex worker (FSW) group. The model stratifies populations further by sex and 5-year age groups (from birth to ages 60 years and above). The model divides the HIV positive population into three disease stages: primary, chronic, and AIDS. For each of the stages, individuals may be diagnosed or undiagnosed. Those diagnosed may initiate 1st line antiretroviral therapy, can progress to a treatment failure group, and a 2nd line ART group.

Finally and most relevantly for this investigation, we divided males into 3 groups according to circumcision status: none, foreskin cut (incorporating men with longitudinal slits or any form of foreskin cut), and circumcised (complete removal of the foreskin). We assumed circumcised men had a 60% reduction in risk of acquiring HIV infection during heterosexual intercourse. For men with a foreskin cut, we assumed a partial level of protection from HIV infection because of exposure of the inner foreskin, and an appearance that can closely resemble MC. After consultation with in-country stakeholders and an assessment of the types of foreskin cutting performed in PNG, we assumed a protective efficacy of 20% for calibration but with a range of 0–60% for our sensitivity analysis.

Model parameter estimates were informed by previous HIV epidemiological estimates, detailed demographic, behavioral, and clinical data, and to reproduce data on the HIV epidemic in PNG from 1990 to 2012. We manually calibrated the model parameters to estimated HIV prevalence, the number of reported cases overall and across age groups, and the number of people who have begun ART producing a “best fit” simulation reconciling all available epidemic and parameter estimates. Table S1 and an associated report [30] provide a detailed description of the model, calibration process and resulting parameter values.

Due to uncertainty in parameter values and epidemic estimates, we generated 200 parameter sets using Latin Hypercube Sampling with the SaSAT software from estimated ranges for key model parameters [31], with parameter ranges and distributions described in Table S1. Monte Carlo filtering was applied to eliminate parameter sets producing simulations with overall prevalence in 1990>0.3% or with prevalence in 2012<0.1% or greater than 3%. We used the resulting 67 simulations to produce all our evaluation results and prediction ranges.

Circumcision and other intervention scenarios were simulated in the model from the end of 2012 to 2032. The proportion of males circumcised each year is given by the coverage target divided by the number of intervention years. At the end of the intervention period, prioritised males returned to being circumcised at birth only at the baseline rate. We obtained results for each of the 67 ensemble simulations and report the mean and range across all simulations.

All calculations were performed with Matlab 2010b (The Mathworks Inc., Natick MA, USA).

## Results

**Figure 2:**
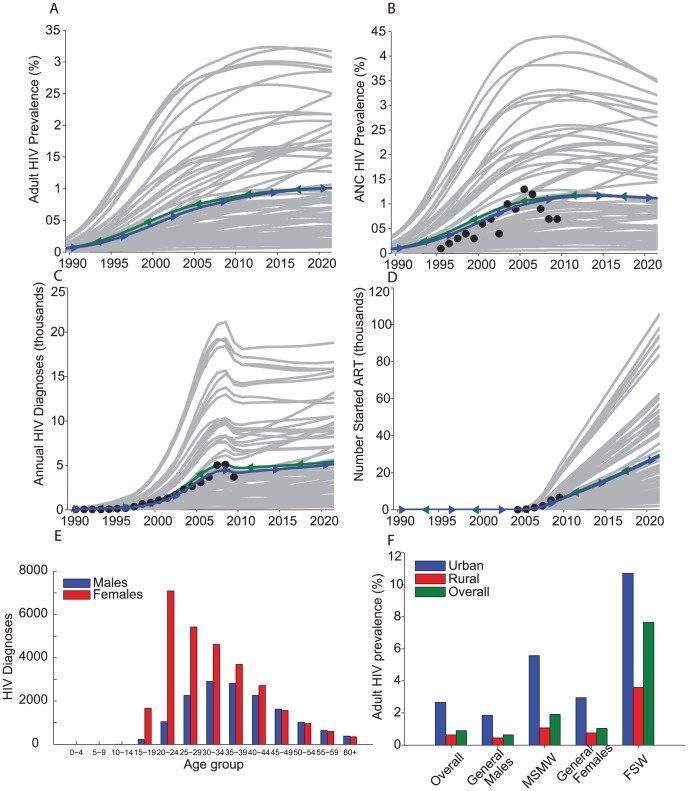
A–D: Data (circle markers), model simulations with estimated parameters (Table S1, blue lines) and model simulations from Latin Hypercube sampling over the parameter ranges (grey lines from 67 selected simulations - simulations were excluded if the overall prevalence was >0.3% in 1990 or <0.1% or >3% in 2012), with the mean of these simulations (green lines), for: A) adult HIV prevalence, B) percentage HIV prevalence in antenatal clinic (ANC) testing, C) annual number of new HIV diagnoses, and D) number (thousands) commencing combination antiretroviral therapy. E) Total male (blue bars) and female (red bars) HIV diagnoses in each age group from the model simulations 1990 to 2010. F) Estimated urban (blue), rural (red), and overall (green) adult HIV prevalence in 2010 among the adult population (Overall), males, men who have sex with men and women (MSMW), females, and female sex workers (FSW).

Simulations of the mathematical model reproduced available data to a substantial degree and produced good estimates for age distributions of male and female HIV diagnoses over the course of the epidemic (Figure 2). Model calculations estimated adult HIV prevalence in 2010 of 0.85%, with higher prevalence in urban (2.7%) compared to rural populations (0.6%). Our estimates of ante-natal clinic (ANC) HIV prevalence of 1.1%, and indeed its dynamics up to this time, coincided well with surveillance data (Figure 2B). The model estimates of numbers of individuals living with HIV infection show a much slower and lower expansion to date and into the future than experienced in sub-Saharan Africa but as indicated by HIV data from PNG (Figure 2A). Our estimated level of 2010 HIV prevalence among FSW in urban regions of 11.4% (rural 3.4%) matches with pooled HIV prevalence from surveys in urban FSW of 11.8% (95% confidence interval (CI) 5.8–17.7%) [23].

### Estimating the impact of increased MC in PNG from 2012 to 2032

We next used the model to estimate the impact of increased MC commenced in 2012 through to the year 2032. We simulated this under two scenarios: the first where only uncut men (approximately 55% of adult males without any form of foreskin cut [14]) underwent MC; and secondly, where both uncut men and those with longitudinal cuts underwent MC. A study of men with longitudinal cuts at 2 universities and 2 rural sites in PNG has shown that 84% of men with a long cut are willing to have their foreskin completely removed if it reduces their risk of HIV acquisition [14]. Hence including this large cohort of adult males is important for a reasonable estimate of the impact of MC programs in this country.

At the national level, the model estimated that circumcising uncut men (those with no existing foreskin cutting) could have a small impact on HIV incidence in PNG. If 30% of all uncut men in PNG were circumcised over a 5-year period 5.5% of new infections could be averted. Approximately 143 MC would be required to avert a single HIV infection regardless of whether 10%, 20% or 30% of uncut men received MC (Table 1). Expanding MC to 80% of uncut men produces little difference with 149 MC per HIV infection averted. The relative benefit was constant partly due to the low percentage of HIV infections averted for this range of interventions (5.5% even with 30% MC). This relatively low rate of averted infections due to MC was directly linked to the low national HIV prevalence, and the high percentage of individuals (45%) with existing foreskin cuts.

**Table 1:** Impact of different circumcision scenarios.

		Infections Averted			Number of males circumcised (Thousands)			Circumcisions per infection averted		
Intervention	Percentage circumcised	10%	20%	30%	10%	20%	30%	10%	20%	30%
**Circumcise uncut men** (excludes men with foreskin cuts)	Percentage of uncut males undergo MC over a 5 year period	4,170 (165–17,600)	7,900 (316–33,200)	11,200 (454–47,100)	195 (104–282)	376 (200–544)	543 (289–786)	141 (14925)	143 (15–931)	144 (15–937)
	Percentage of uncut 15 to 19 year olds undergo MC over a 5 year period	611 (23–2,540)	1,190 (44–4,940)	1,740 (65–7,200)	21(11–30)	41(22–59)	60(32–87)	109 (10–644)	109 (10–644)	109 (10–643)
	Percentage of uncut 15 to 24 year olds undergo MC over a 5 year period	1,450 (53–6,110)	2,790 (102–11,800)	4040 (149–17,000)	39(21–56)	75(41–109)	109 (59–158)	85 (8–533)	86 (8–535)	86 (8–536)
	Percentage of uncut 15 to 29 year olds undergo MC over a 5 year period	2,300 (83.7–9,870)	4,400 (161–18,800)	11,200 (454–47,100)	54 (29–78)	104 (56–151)	543 (289–786)	74 (7–486)	75 (7–488)	144 (15–937)
	Percentage of uncut 15 to 34 year olds undergo MC over a 5 year period	2,860 (106–12,300)	5,470 (204–23,400)	7,830 (295–33,500)	67 (36–97)	128 (69–186)	186 (100–270)	73 (7–489)	73 (7–492)	74 (7–494)
	Percentage of uncut 15 to 24 year olds in urban areas undergo MC over a 5 year period	392 (5–1650)	946 (11–4620)	1,370 (16–6,700)	5 (3–7)	12 (6–20)	18 (8–29)	61 (3–857)	54 (3–781)	54 (3–780)
**Circumcise men with or without a longitudinal foreskin cut**	Percentage of uncut males and those with foreskin cut undergo MC over a 5 year period	6,480 (343–22,600)	12,200 (652–42,200)	17,200 (930–59,300)	367 (347–383)	707 (668–737)	1,020 (965–1,060)	171 (16–1,080)	175 (16–1,090)	178 (17–1,110)

For each scenario the mean and range of the simulation ensemble are presented. Mean (and Latin Hypercube Sampling ranges) of numbers of HIV infections averted, numbers of adult males circumcised, and numbers of circumcisions carried out to avert one infection in the period 2012–2032 for each circumcision intervention scenario.

Prioritizing circumcision uptake to men aged between 15 and 34 years will result in a better cost-benefit ratio of 73 MCs required to avert a single HIV infection (Table 1). The 30 to 34 year age group coincides with the largest number of recorded male HIV diagnoses in 2008 [32], as well as the highest number of male diagnoses over the course of the epidemic from our model (Figure 2E). The MC per infection averted ratio is further improved by concentrating MC to 15 to 24 year old men in urban areas, the age at which most foreskin cutting is taking place [14]. This results in 54 MC per infection averted once MC is provided to 20% or more of this age group (Table 1).

### Estimated impact of penile cutting

**Figure 3:**
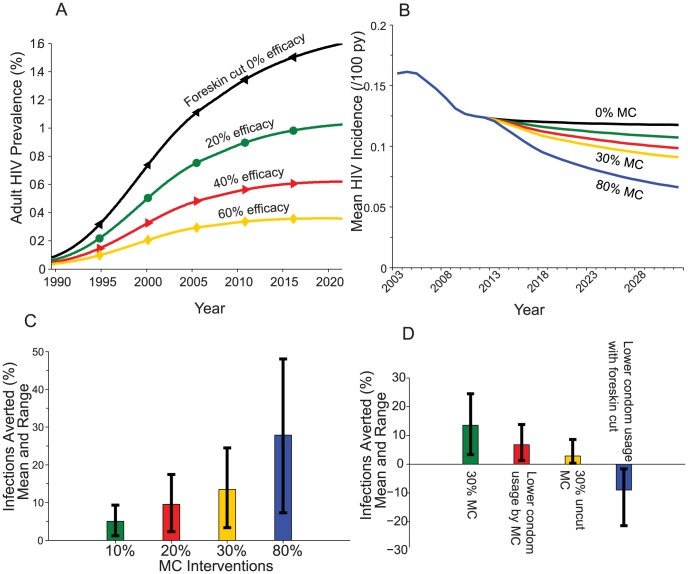
A) HIV prevalence if foreskin-cutting efficacy is fixed to 0%, 20%, 40%, and 60%. The lines show the mean prevalence from the 67 selected simulations with the foreskin-cutting efficacy fixed to a particular value. B) Impact on HIV incidence if males who are uncut, and those who have a longitudinal cut, undergo MC during a five-year circumcision program. No MC (black line in A), 10% (green), 20% (red), 30% (yellow), and 80% (blue) of men undergo MC; C) percentage infections averted by 2032 by scenarios in B); and D) Percentage infections averted if a MC program that circumcises 30% of uncut males over a 5 year period (30% MC, green column) results in subsequent risk compensation: 30 percentage point reduction in condom use in this group (red column); 30% uncut males undergoing foreskin cutting instead (yellow column); or all males with foreskin cutting reducing their condom use by 30 percentage points believing they are protected (blue column).

Although MC provides approximately 60% reduction in the risk of acquiring HIV through heterosexual contact, the protective effect of foreskin cutting in PNG is unknown. We expect the protective efficacy of these practices to depend on the extent of inner foreskin exposure. This can vary greatly depending on the length of the dorsal slit [15], [16]. In the calculations above, we assumed non-MC foreskin cuts reduced the likelihood of HIV acquisition by the male partner in heterosexual intercourse by 20%. Assuming these non-MC forms in 45% of men had no protective effect, and repeating simulations from the start of the epidemic in PNG up to the present would have led to higher HIV prevalence among 16 to 49 year olds of 1.3% in 2012, compared to the above model calculations of 0.9% with 20% protection, and 0.3% HIV prevalence if foreskin cutting exhibited 60% efficacy, the same as MC (Figure 3A). Hence any protective effect of cutting will have affected current HIV prevalence in the population and also influence any potential outcome of a population level MC intervention (Figure 3B,C). If a large proportion of men have a foreskin cut (and assuming an average protective effect of 20%) and MC is provided to both uncut men and those with longitudinal cuts (resulting in removal of the remaining foreskin) then approximately 175 MC would be required to avert a single HIV infection (Table 1). If uncut males only are provided MC then the number of MC per infection averted would decrease to approximately 143 since we assume those with foreskin cuts already have some protection.

### Benefits of increased MC versus condoms

For comparison, we also estimated the impact of increased levels of condom usage in the general community, or for FSW and their male clients. In PNG, knowledge of condoms is generally high but the use of condoms in the general population tends to be low, with many studies reporting a high proportion of people having never used a condom including those that are HIV positive. Self-reported values for condom use at last act from a number of surveys of males and females in the general population show an increasing usage over time for casual partnerships, to a level of approximately 40% in 2008 in urban settings and 20% in rural regions, with roughly half as frequent condom usage in regular partnerships [29], [33]. Frequency of condom usage between FSW and their clients, as well as with their regular partners, has also increased over time reaching approximately 70% and 40% respectively in urban regions [29], [33]. We assume condom usage in rural areas to be half these values because of lower condom availability and accessibility, less access to health services and health campaigns, lower education attainment, and more conservative religious influences.

Increasing condom usage from 2012 by an absolute amount of 10% (from 20% to 30% in urban regions) in regular heterosexual contacts would avert 18,400 infections (Table 2) almost twice the impact of circumcising 30% of uncut males (11,200 infections averted, Table 1). Circumcising 30% of males would require half a million circumcisions to be performed over 5 years (Table 1). Increasing condom usage by male clients of FSW will also yield superior results to MC (Table 2). The impact of MC delivered to 20% of uncut males (3,063 infections averted) was roughly equivalent to the effect achieved by increasing condom usage in casual partnerships by 30% (5,890 infections averted, Table 2).

**Table 2:** Number of HIV infections averted with increased condom usage from 2012 to 2032, or in combination with MC.

Condom use intervention		Infections Averted no MC program	Infections Averted with 20% of uncut males undergo MC over a 5 year period
**Increase in condom usage by clients/partners of FSW in commercial and casual partnerships**	10 percentage point increase in condom usage	13,100 (375–40,100)	19,600 (715–59,100)
	20 percentage point increase in condom usage	23,300 (708–70,800)	28,800 (1,030–86,200)
	30 percentage point increase in condom usage	31000 (1,000–93,800)	35,800 (1,300–107,000)
**Increase condom usage in casual partnerships**	10 percentage point increase in condom usage	2,010 (104–10,600)	9,690 (459–42,400)
	20 percentage point increase in condom usage	3,970 (206–20,800)	11400 (558–51,200)
	30 percentage point increase in condom usage	5,890 (307–30,600)	13,200 (654–59,700)
**Increase condom usage in regular partnerships**	10 percentage point increase in condom usage	18,400 (1,410–85,600)	24,500 (1,660–106,000)
	20 percentage point increase in condom usage	33,900 (2,670–14,8000)	38600 (2,870–160,000)
	30 percentage point increase in condom usage	47,000 (3,800–193,000)	50,600 (3,970–200,000)

For each scenario the mean and range of the simulation ensemble are presented.

### Risk compensation following MC

With any intervention there is a concern that individuals will modify their behavior to the point where any real or perceived benefits of that intervention are lost. For each 10 percentage point decrease in condom usage for the 30% of uncut males who undergo MC, there is an approximate 10% decrease in number of infections averted (Table 3, Figure 3D). If this decrease in condom usage also carries over to individuals with existing foreskin cuts, then a 10% decrease in condom use by all individuals with foreskin cuts would reverse the 11,200 infections averted with the MC program and lead to an increase of 4,120 HIV infections from the current situation. A summary of all interventions is included in Table 4.

**Table 3:** The number of HIV infections averted in the period 2012–2032 when risk compensation or behaviour change occurs after the introduction of MC.

Change in behaviour scenario due to knowledge or implementation of MC		Infections Averted
**Uncut men have longitudinal penile cut (at an assumed average 20% efficacy against HIV acquisition) instead of MC in the belief it is effective**	10% of uncut males undergo foreskin cutting over a 5 year period	1,280 (−24–8,380)
	20% of uncut males undergo foreskin cutting over a 5 year period	2,460 (−47–16,000)
	30% of uncut males undergo foreskin cutting over a 5 year period	3,530 (−68–22,800)
**Decrease in condom use in men who undergo MC (assuming 30% undergo MC over 5 year period)**	10 percentage point reduction in condom use each year by men who have MC	10,300 (423–44,700)
	20 percentage point reduction in condom use each year by men who have MC	9,310 (353–42,300)
	30 percentage point reduction in condom use each year by men who have MC	8,330 (8–39,900)
**Decrease in condom use in men with foreskin cutting (because they believe foreskin cutting lowers their risk)**	10 percentage point reduction in condom use each year by men who have a cut foreskin	−4,120 (−14,900, −129)
	20 percentage point reduction in condom use each year by men who have a cut foreskin	−8,510 (−30,600, −261)
	30 percentage point reduction in condom use each year by men who have a cut foreskin	−13,200 (−47,100, −394)

Negative values indicate an increase in infections relative to baseline.

For each scenario the mean and range of the simulation ensemble are presented (negative values reflect an increase in infections).

**Table 4:** Summary table of interventions.

Scenario	Targeted Groups	Mean HIV infections averted; MC per HIV averted where relevant, at the 20% MC level
**MC**	Uncut men	7,900; 143
	Uncut 15–19 year olds	1,190; 109
	Uncut 15–24 year olds	2,790; 86
	Uncut 15–29 year olds	4,400; 75
	Uncut 15–34 year olds	5,470; 73
	Urban uncut 15–24 year olds	946; 54
	All males	12,200; 175
		**Mean HIV infections averted**
**Increased condom usage**	FSW clients/partners	10% increase: 13,100 (0% MC); 19,600 (20% MC)
	Casual partnerships	10% increase: 2,010 (0% MC); 9,690 (20% MC)
	Regular partnerships	10% increase: 18,400 (0% MC); 24,500 (20% MC)
**Increased foreskin cutting**	Uncut men	10% increase: 1,280
**Decreased condom usage**	Men with MC	10% decrease with 30% MC: 10,300
	Men with foreskin cut	10% decrease: −4,120

## Discussion

We and others have shown that increased MC in high prevalence countries, where the main mode of HIV transmission is through heterosexual contact, can lead to sizeable reductions in HIV incidence [4]–[6]. It is therefore understandable that even in countries with regions of moderate HIV prevalence, MC would be of considerable interest to both health authorities and indeed to individuals. PNG with an estimated HIV prevalence of approximately 1% of the adult population, the country with the highest HIV prevalence in Oceania and second highest in Asia-Pacific [11], fits into this category.

Against this background and the prevalent foreskin cutting practices, we estimated that a nationwide MC program, providing a complete removal of the foreskin through a circumferential cut, would have limited benefits in PNG. Whereas MC in sub-Saharan Africa was calculated to avert 1 HIV infection per 5 to 15 MC [6], our calculations determined that on average 143 MC would need to be performed to avert a single HIV infection in PNG. The most effective MC intervention concentrated delivery to 15 to 24 year old urban men, requiring 54 MC per infection averted. Targeting young men attending Voluntary Counseling and Testing (VCT) and STI clinics in areas of the country with the highest HIV prevalence and lowest longitudinal foreskin cutting may be even more effective.

The efficacy of longitudinal dorsal slit forms of foreskin cutting in inhibiting HIV acquisition during heterosexual acts is uncertain. As 45% of men in PNG had dorsal slits this uncertainty had a large impact on estimates of HIV prevalence, ranging from 0.3% in 2012 if foreskin cuts were as effective as MC up to 1.3% if there was no effect (Figure 3A). This also affected the number of MC required to avert an infection with a better cost-effectiveness ratio if foreskin cuts do not inhibit HIV acquisition. This difference in effect, dependent on the level of protection provided by the dorsal slit, is understandable since where there is a high background level of foreskin cutting, any benefit this provides will lower HIV prevalence, make HIV infections less frequent, and therefore require more MC to avert each new HIV infection. The protective effect of foreskin cutting could potentially be having a significant impact on HIV prevalence in PNG and therefore may affect any additional impact of a MC program in the future. Its impact on likelihood of HIV acquisition is the subject of further investigation by our group. Nevertheless, any advantages it may provide in reducing HIV acquisition might be outweighed by complications associated with it being performed under unsafe conditions.

Factors not taken into account in these simulations were any benefits provided by MC, or of dorsal slits for that matter, in terms of reduced risk of attaining other STI. A longitudinal study, performed by our group, of sexual health clinic attendees in PNG determined that men with a dorsal slit were significantly less likely to become infected with syphilis during the 50 week follow-up period (but more likely to acquire chlamydia infection [34]). This is consistent with other research findings that MC can confer protection against syphilis [35], and other STI [36].

We found increased and consistent condom usage, an intervention with potentially fewer initial infrastructure requirements, produces a far greater benefit than MC. On the other hand evidence from PNG and other settings indicate it is unlikely risk compensation, in terms of lower condom usage, would completely offset reductions in HIV incidence following an MC program. A recent study in PNG found no difference in condom use at last sex between men with MC ("round cut"), long cut and no cut (35%, 32%, and 33% respectively) [14]. Similarly there were no differences in condom use with non-spousal partners between circumcised and uncircumcised men in a South African township that was the site of a randomized controlled trial of MC [3].

This complex background of foreskin cutting, uncertainties in the protective effects of such cuts against HIV acquisition, and the moderate levels of HIV prevalence in PNG all make for a multifaceted environment. Despite these uncertainties we found that contrary to the recognized benefits of providing MC in sub-Saharan Africa [1], [2], an extensive MC intervention uniformly across PNG was not advisable requiring approximately 143 circumcisions to avert a single HIV infection. However, expanding the availability of MC in certain locations and populations in PNG warrants further investigation, especially for areas with higher prevalence such as the National Capital District, and the Enga and Western Highlands provinces, and in the younger age groups where MC was estimated to be most effective in reducing new HIV infections. There may also be benefits of a clinic-based medical MC program to reduce the risk of complications associated with longitudinal foreskin cuts and other forms of foreskin cutting conducted in non-clinical settings. Nevertheless, we found increasing condom usage in PNG would have a greater population-level benefit than MC under current conditions.

## Supporting Information

Table S1 **Summary of model parameters and uncertainty ranges.** For each parameter, we estimated a value by calibrating the model to represent the estimated HIV prevalence, the number of reported cases overall and across age groups, and the number of people who have begun ART. Uncertainty ranges for parameters are based on available data or are assumed where no data is available. For beta distributed uncertainty ranges, the estimated value represents the mean value. We assumed wide uncertainty ranges for values that are particularly important for HIV transmission, in particular the circumcision and penile cutting parameters.
(DOC)
