# Supplementary material for: Impact of Male Circumcision on the HIV Epidemic in Papua New Guinea: A Country with Extensive Foreskin Cutting Practices
Source: PLoS One. 2014 Aug 11;9(8):e104531. doi: 10.1371/journal.pone.0104531 (PMC4128698; doi:10.1371/journal.pone.0104531)
Supplement: Table S1 — Summary of model parameters and uncertainty ranges. For each parameter, we estimated a value by calibrating the model to represent the estimated HIV prevalence, the number of reported cases overall and across age groups, and the number of people who have begun ART. Uncertainty ranges for parameters are based on available data or are assumed where no data is available. For beta distributed uncertainty ranges, the estimated value represents the mean value. We assumed wide uncertainty ranges for values that are particularly important for HIV transmission, in particular the circumcision and penile cutting parameters. (DOC) [file pone.0104531.s001.doc]

# Impact of male circumcision on the HIV epidemic in Papua New Guinea: a country with extensive foreskin cutting practices

Richard T. Gray^1^, Andrew Vallely^1,2^, David P. Wilson^1^, John Kaldor^1^, David MacLaren^3^, Angela Kelly-Hanku ^2,4^, Peter Siba^2^, John M. Murray^5, †^ on behalf of the Male Circumcision Acceptability and Impact Study (MCAIS) team)

^1^ The Kirby Institute, University of New South Wales, Sydney, NSW 2052, Australia;

^2^ Sexual and Reproductive Health Unit, Papua New Guinea Institute of Medical Research, Goroka, Papua New Guinea;

^3^ School of Medicine and Dentistry, James Cook University, Cairns QLD, Australia;

^4^ International HIV Research Group, School of Public Health and Community Medicine, University of New South Wales, Sydney, NSW 2052, Australia;

^5^ School of Mathematics and Statistics, University of New South Wales, Sydney, NSW 2052, Australia

# Supplementary Table S1

**Table S1 - Summary of model parameters and uncertainty ranges**.

For each parameter, we estimated a value by calibrating the model to represent the estimated HIV prevalence, the number of reported cases overall and across age groups, and the number of people who have begun ART. Uncertainty ranges for parameters are based on available data or are assumed where no data is available. For beta distributed uncertainty ranges, the estimated value represents the mean value. We assumed wide uncertainty ranges for values that are particularly important for HIV transmission, in particular the circumcision and penile cutting parameters.

| Parameter | Estimated  Values | Time  Varying | Age  Varying | Uncertainty  Range | Distribution | Footnote |
| --- | --- | --- | --- | --- | --- | --- |
| **Demographic Parameters** | | | | | | |
| Percentage of urban males that are MSMW | 6% | No | No | 1-10% | Uniform | 1 |
| Percentage men in rural areas that are MSMW | 4% | No | No | 0-10% | Beta | 1 |
| Percentage of females in urban areas that are FSW | 5% | No | No | 2-8% | Uniform | 2 |
| Percentage of females in rural areas that are FSW | 1% | No | No | 0-5% | Beta | 2 |
| Percentage of FSW who stop engaging in sex work in year | 5% | No | No | 0-10% | Uniform | 3 |
| Percentage of population that migrates from rural to urban areas each year | 1% | No | No | 0-5% | Beta | 4 |
| **HIV Transmission Parameters** | | | | | | |
| Per-act reference transmission probability from chronically infected females to males during unprotected sexual intercourse | 0.00052 | No | No | 0.0001-0.0014 | Beta | 5 |
| Per-act reference transmission probability from chronically infected males to females during unprotected sexual intercourse | 0.002 | No | No | 0.0006-0.003 | Beta | 5 |
| Per-act male to male  reference transmission probability | 0.009 | No | No | 0.0024-0.0276 | Beta | 5 |
| Prevalence of sexually transmitted infections other than HIV in each population group | Varies across groups | No | No | ±25% | Uniform | 6 |
| Multiplicative factor for increased HIV transmission probability due to the presence of STIs | 5 | No | No | ±25% | Uniform | 7 |
| **Sexual Behaviour Parameters** | | | | | | |
| Number of casual and regular partners per year – non MSMW | Varies across groups | Yes | Yes | ±25% | Uniform | 8 |
| Number of casual males partners per year for MSMW | 1-2 | Yes | Yes | 0-10 | Beta | 9 |
| Number of regular male partners per year for MSMW | 0.075-0.1 | Yes | Yes | 0-3 | Beta | 9 |
| Per act probability of condom usage | Varies across groups | Yes | Yes | ±25%  (max 100%) | Uniform | 10 |
| Acts of sexual intercourse per casual partnership | 1 | No | No | ±25% | Uniform | 11 |
| Acts of sexual intercourse per regular partnership | 100 | No | No | ±25% | Uniform | 11 |
| **Clinical Parameters** | | | | | | |
| Proportion of HIV+ individuals with primary or chronic HIV infection that are diagnosed with HIV each year | Varies across groups | Yes | Yes | ±25% | Uniform | 12 |
| Proportion of people with AIDS diagnosed each year. | Varies across groups | Yes | No | ±25% | Uniform | 13 |
| Proportion of HIV infected individuals with diagnosed AIDS that begin first line ART treatment each year | Calibrated to match data on number that have started ART | Yes | No | ±25% | Uniform | 14 |
| Proportion of people on first line ART in urban areas that experience treatment failure each year | 10-20% | No | No | ±25% | Uniform | 15 |
| **Circumcision & Penile Cutting parameters** | | | | | | |
| Percentage of urban and rural males that are circumcised | 5% | No | No | 0-15% | Beta | 16 |
| Percentage of urban and rural males with longitudinal penile cutting | 45% | No | No | 20-70% | Uniform | 16 |
| Reduction in HIV acquisition for circumcised general males and MSMW through vaginal intercourse | 60% | No | No | 50-70% | Uniform | 17 |
| Reduction in HIV acquisition for general males and MSMW with foreskin cutting through vaginal intercourse | 20% | No | No | 0-60% | Beta | 18 |
| 1: The proportion of men that are MSMW is based on data from surveys but is highly uncertain. We assume a higher percentage in urban areas to reflect levels similar to MSM populations in high-income countries.  2: It is difficult to separate female sex workers from the general female population due to the high levels of transactional sex that occur throughout PNG. The values are based on data from surveys with assumed ranges reflecting this uncertainty. We assume a higher percentage in urban populations.  3: The proportion of FSW who stop sex work and return to the general female population was calibrated to maintain the proportion of females who are sex workers. FSW older than 45 are assumed to stop sex work.  4: Population movement within PNG is very common with people travelling regularly across the country. However, there is no quantitative data available on population movement in PNG. Due to this uncertainty, we assumed that 1% of people in rural areas move to urban areas each year with a large percentage range. This value is fixed and the same for each age group. Migration from urban to rural areas is set automatically in the model so that the overall percentage of people in urban areas is fixed over time.  5: The per-act contact rates for male to female, female to male, and male to male transmission are based on numerous studies and represent the probability of HIV transmission from an infected partner who is in the chronic infection stage and not on ART during a sexual act with neither partner infected with other STIs . The values we use here have been calibrated to match the HIV epidemiology data for PNG while remaining in the reported confidence intervals for the per act transmission probabilities. One exception to this is that we allow the male to female transmission probability to be higher to take into account the level of anal sex and sexual violence that are present in PNG .  6: The prevalence of STIs in each population group have been calibrated to the HIV epidemic based on available survey data. We assume a 25% uncertainty for all population groups.  7: This multiplicative increase in HIV transmission probability if one partner in a discordant partnerships has an STI is based on the estimates for genital ulcer disease in the Boily et al. meta-analysis . We assume a 25% uncertainty for all population groups.  8: The numbers of sexual partners each population group has each year were calibrated to the PNG HIV epidemic and change over time with the same relative reduction in partners. A small reduction in partner numbers is assumed for individuals with AIDS or who have been diagnosed with HIV due to illness and education/counseling.  9: Despite surveys showing MSMW can have high levels of sexual activity there have been low numbers of recorded transmissions attributed to homosexual contact and there is a relatively low HIV prevalence in MSMW. This means to calibrate the model we had to assume the average number of male partners MSMW have each year is low reflecting infrequent sexual intercourse between men on average. Given this is at odds with other MSM populations a wide range in the number of partnerships for MSMW. We assumed urban MSMW have twice the number of sexual partners than rural MSMW to reflect the presence of a higher sexually active homosexual population in urban areas.  10: Condom usage varies across population groups depending on the type of partnership and was informed by available data. Data suggests that condom use has increased over time.  11: These values are based on standard assumptions used in the HIV modeling literature. For regular partnerships the 100 acts corresponds to the assumed number of acts in a long-term regular partnership each year.  12: Diagnosis of HIV+ individuals assumed that rural testing rates are less than those for urban populations due to lower access to medical services. The relative change in testing rate from 1990 to 2010 is the same for each population and age group. There is no variation with age in testing rates assumed for general males, MSMW, or FSW populations. Younger general females have higher testing rates than older females due to ANC testing. The parameter values were calibrated to match the cumulative number of diagnoses in each age group. All populations are assumed to have the same relative increase in testing over time to match the rise in recorded diagnoses in PNG. General males and MSMW are assumed to have the same testing rates overall while FSW are assumed to have a higher testing rate than general females due to their higher risk of acquiring HIV and to reflect available testing data. We assume a 25% uncertainty.  13: Individuals with AIDS have a higher testing rate due to the presence of clinical symptoms and was assumed to be the same for each population group. Urban populations have a higher rate than rural populations due to better access to medical services. Diagnoses rates for people with AIDS are assumed to be much higher than for the rates during primary and chronic HIV infection and are calibrated so that simulated annual diagnoses matches the recorded diagnoses in PNG overall. We assumed a 25% uncertainty range.  14: ART only became available in PNG at the end of 2003, since then HIV infected individuals have been given treatment based on WHO guidelines. Despite expected differences between rural and urban areas due to logistical problems we assume the same rate of ART initiation for both urban and rural areas (with the differences between urban and rural areas contained in the AIDS diagnoses rate). We assumed a 25% uncertainty range.  15: There is no data available on HIV treatment failure in PNG. These values and ranges are assumed based on available clinical data . All population and age groups have the same failure rate with urban areas having half the failure rate of rural areas reflecting better access to ART services. We assumed a 25% uncertainty range.  16: Men in PNG are likely circumcised after birth or after they become sexually active but it is assumed that men undergo these procedures prior to becoming sexually active. The proportion of men that have penile cutting or are circumcised is particularly important for evaluating MMC so we have assumed a wide range for these proportions.  17: The protective effect of complete foreskin removal is estimated from the results of a number of random control trials evaluating the impact of circumcision of HIV transmission .  18: For penile cutting and the partial removal of the foreskin there is no data available on the relative effectiveness in reducing the risk of HIV acquisition but it is likely to vary widely depending on the level of foreskin removal. Based on discussions with local stakeholders and experts we assumed a 20% reduction for men who have any penile cutting with a wide uncertainty range from no protection (0%) to the same protection as circumcision (60%). | | | | | | |

## References

1. Attia S, Egger M, Müller M, Zwahlen M, Low N. Sexual transmission of HIV according to viral load and antiretroviral therapy: systematic review and meta-analysis. *AIDS (London, England)* 2009,**23**:1397-1404.

2. Boily M-c, Baggaley RF, Wang L, Masse B, White RG, Hayes RJ*, et al.* Heterosexual risk of HIV-1 infection per sexual act: systematic review and meta-analysis of observational studies. *Lancet Infectious Diseases* 2009,**9**:118.

3. Jin F, Jansson J, Law M, Prestage GP, Zablotska I, Imrie JCG*, et al.* Per-contact probability of HIV transmission in homosexual men in Sydney in the era of HAART. *AIDS (London, England)* 2010,**24**:907-913.

4. Quinn T, Wawer M, Sewankambo N, ERWADDA DS, LI CH, ABWIRE-MANGEN FW*, et al.* Viral load and heterosexual transmission of human immunodeficiency virus type 1. *New England Journal of Medicine* 2000,**342**:921-929.

5. Vittinghoff E, Douglas J, Judson F, McKirnan D, MacQueen K, Buchbinder SP. Per-contact risk of human immunodeficiency virus transmission between male sexual partners. *American journal of epidemiology* 1999,**150**:306-311.

6. Wawer MJ, Gray RH, Sewankambo NK, Serwadda D, Li X, Laeyendecker O*, et al.* Rates of HIV-1 Transmission per Coital Act, by Stage of HIV-1 Infection, in Rakai, Uganda. *The Journal of infectious diseases* 2005,**191**:1403-1409.

7. Wilson D, Law M, Grulich A, Cooper D. Relation between HIV viral load and infectiousness: a model-based analysis. *The Lancet* 2008,**372**:314-322.

8. Gray RT, Maibani-Michie G, Lupiwa T, Yeka W, Claire R, Wilson D. A Behavioural and Biological Survey of HIV and STIs in Papua New Guinea 2003-2008 - Draft. In; 2010.

9. Jenkins C. Cultures and Contexts Matter: Understanding and Preventing HIV in the Pacific. In: *Contexts*; 2007.

10. King E, Lupiwa T. A Systematic Literature Review of HIV and AIDS in Papua New Guinea 2007-2008. In. Port Moresby; 2009.

11. Smith CJ, Phillips AN, Hill T, Fisher M, Gazzard B, Porter K*, et al.* The rate of viral rebound after attainment of an HIV load <50 copies/mL according to specific antiretroviral drugs in use: results from a multicenter cohort study. *The Journal of infectious diseases* 2005,**192**:1387-1397.

12. Auvert B, Taljaard D, Lagarde E, Sobngwi-Tambekou J, Sitta R, Puren A. Randomized, controlled intervention trial of male circumcision for reduction of HIV infection risk: the ANRS 1265 Trial. *PLoS medicine* 2005,**2**:e298.

13. Bailey RC, Moses S, Parker CB, Agot K, Maclean I, Krieger JN*, et al.* Male circumcision for HIV prevention in young men in Kisumu, Kenya: a randomised controlled trial. *The Lancet* 2007,**369**:643-656.

14. Gray RH, Kigozi G, Serwadda D, Makumbi F, Watya S, Nalugoda F*, et al.* Male circumcision for HIV prevention in men in Rakai, Uganda: a randomised trial. *The Lancet* 2007,**369**:657-666.
